# The effect of gelatin as pore expander in green synthesis mesoporous silica for methylene blue adsorption

**DOI:** 10.1038/s41598-022-19615-5

**Published:** 2022-09-10

**Authors:** Maria Ulfa, Didik Prasetyoko, Wega Trisunaryanti, Hasliza Bahruji, Zahra Ayu Fadila, Novia Amalia Sholeha

**Affiliations:** 1 Chemistry Education Study Program, Faculty of Teacher Training and Education, Sebelas Maret University, Jl. Ir. Sutami 36A, Surakarta, 57126 Indonesia; 2 Department of Chemistry, Faculty of Science and Data Analytics, Institut Teknologi Sepuluh Nopember, Keputih, Sukolilo, Surabaya, 60111 Indonesia; 3 Department of Chemistry, Faculty of Science, Gadjah Mada University, Sekip Utara Sleman, Indonesia; 4 Centre of Advanced Material and Energy Sciences, University Brunei Darussalam, Jalan Tungku Link, 1410 Brunei Darussalam; 5 College of Vocational Studies, Bogor Agricultural University (IPB University), Jalan Kumbang No. 14, Bogor, 16151 Indonesia

**Keywords:** Environmental sciences, Chemistry, Materials science, Nanoscience and technology

## Abstract

Mesoporous silica NSG had been synthesized while employing gelatin as a natural template to successfully increase the particle size and expand the pore diameter of NSG. All silica samples exhibited a similar XRD pattern with a broad peak centred at 2θ = 22.9°, as the characteristic of amorphous silica. FTIR results showed that the reduction of Si–O–Si symmetric stretching vibrations at 1075 cm^−1^ was due to the use of a high percentage of gelatin. Moreover, TEM analysis displayed the mesoporous channels in the form of a honeycomb structure with a diameter of ± 6 nm. Gelatin enhanced the surface area of silica from 467 to 510 m^2^/g, the pore volume from 0.64 to 0.72 cc/g and expanded the pore diameter from 3.5 nm to 6.0 nm. The expansion of the ordered mesopores with the increase of P123: gelatin ratios was elucidated by the pore size distribution. The adsorption capacity of methylene blue (MB) was improved on mesoporous silica with an expanded pore dimension to give 168 mg/g adsorption capacity within 70 min.

## Introduction

Mesoporous silica is widely applied as adsorbents for waste removal^1^, carrier in drug delivery system^2^, as catalyst supports^3^ and material for energy storage^4^. Synthesis of silica with high surface area and ordered pore structure is beneficial for a variety of applications as it improves diffusion of molecule reactant. The main concern circumventing the safety of the synthesis procedures is to reduce the use of harmful reagents. Green synthesis aims to minimize the use of synthetic chemicals that are toxic and harmful to the environment^5,6^. Utilization of biomass waste as silica sources such as rice husk ash and palm residue reduced the reliance on synthetic chemicals^7,8^. Direct oxidation of silicon-rich biomass waste often produced silica with a low surface area and a non-uniform structure^7,9^ restricting the performance as adsorbent of large molecules such as ibuprofen, dibenzothiophene and methylene blue^10–12^. The properties of silica obtained from pyrolysis of biomass were improved when the process was combined with acid and alkaline extraction of silica from biomass prior to pyrolysis^13–16^. However, extraction of silica from fly ash biomass using NaOH only yielded on average of 0.001% of silica from the amount of biomass used^1^.

Synthesis of porous material using an environmentally friendly organic–inorganic pore directing agent is also seen as a pathway to suppress the instability of natural material while simultaneously reduce the use of synthetic inorganic materials^9,11,17^. Tailored synthesis of silica via sol–gel, precipitation, chemical vapour deposition or microemulsion methods produced silica with uniform structures and morphologies^8,15,18^. The sol–gel method is generally involved the utilization of surfactants or pore-directing molecules in the form of synthetic block copolymers to mould the shape, size and porosity^1,15^. However, the application of synthetic surfactant is hampered by its expensive price, non-biodegradable properties, and non-sustainable in particular for large-scale production^15,19,20^. Natural pore-directing molecules from gelatin, gum Arabic and starch have been reported in the synthesis of porous silica^21–23^. Gelatin is obtained from hydrolysis of animal skin and bone waste which has high sustainability due to the abundant sources and the feasibility of production. The stability of gelatin and the interaction between the functional group of gelatin and various metal precursor allowed the formation of well-defined crystal structures even after calcination at high temperatures^24^. Previous research on a hybrid system consisting of synthetic and natural pore-directing molecules was able to produce high surface area carbon microspheres as a result of the combination of gelatin with Pluronic F127^6^. In addition to Pluronic F127, several synthetic templates such as Pluronic P123, CTAB, and CTAOH can control porosity based on their molecular weight, chain length and functional groups^19,25,26^. These synthetic pore templates have succeeded in producing porous materials such as MCM-41, MCM-48, SBA-15 and KIT^2,27–29^.

Amine functional groups in gelatin produced electrostatic interaction with silicate ions, capable of directing molecular arrangement for the production of materials with enhanced physicochemical properties^11,30,31^. In this study, silica nanoparticles with well-defined mesopores were synthesized using gelatin and Pluronic P123 templates. Gelatin is proposed to act as a natural pore directing agent and strengthen the stability of P123 to hold the structure following calcination at high temperatures. This study will observe the effect of gelatin in expanding the pore structure of mesoporous silica. The structural properties of mesoporous silica will be characterized by XRD, FTIR, nitrogen adsorption–desorption and SEM EDX, TEM and its performance will be applied as an adsorbent for methylene blue.

## Experimental procedures

### Synthesis of mesoporous silica-gelatin (NSG)

Surfactant P123 and gelatin were dissolved in concentrated hydrochloric acid (37% v/v) and distilled water then stirred at 500 rpm for 3 h with a temperature of 40 °C. TEOS as a silica source was added and stirred until homogeneous. The molar ratios of the chemicals used in this study were 1 TEOS: 0.017 P123: 5.68 HCl: 197 H_2_O. The mixture was continuously stirred at the same temperature for another 24 h. The procedure was repeated with a different ratio of P123: gelatin and labelled as NSG-x%, where x referred to the gelatin concentration given in Table 1.

**Table 1:** The sample was labelled according to the ratio of P123 and gelatin.

Notation of sample	P123: gelatin (w/w)
NS	1:0.00
NSG-1%	1:0.01
NSG-2%	1:0.02
NSG-4%	1:0.04

The mixture was poured into a Teflon line autoclave and heated in an oven for 24 h at 100 °C. The white precipitate obtained was filtered using a Buchner funnel and washed with 200 mL of distilled water. The resulting solid is dried for 24 h at 100 °C and grounded to produce a fine powder. To remove the templates, the solid is calcined at 550 °C for 5 h and placed in a vial at room temperature.

### Methylene blue (MB) adsorption

Methylene blue (MB) solution at 50 mg/L was prepared by dissolving methylene blue in deionised water. Then 50 mg of adsorbent was mixed with 200 mL of MB solution and stirred at 200 rpm for 20 min at room temperature. The concentration of MB solution was determined at different contact times by extracting 3 ml of the solution for every 5 min intervals. The effect of adsorbent weight and initial concentration of MB solution were observed using different adsorbent weights (5,10,25, 50, 70 mg) while the initial MB concentration was varied at 10, 20, 50, 100, mg/L. The concentration of MB was determined using UV spectrophotometer (Shimadzu-230PI). The maximum absorption wavelength of methylene blue was recorded at 664 nm. The adsorption capacity of methylene blue on silica sample is calculated using the following equation:

1 $${\text{q}}_{{\text{e}}} = \left( {{\text{C}}_{{\text{o}}} - {\text{C}}_{{\text{e}}} } \right)\frac{{\text{w}}}{{\text{m}}}{\text{q}}_{{\text{e}}}$$

where Co is the initial concentration and Ce is the equilibrium concentration (mg/L) of MB in the bulk phase. The symbol *w* represents the amount of liquid phase (L) while *m* is the weight of the adsorbent (g). The adsorption kinetics model was calculated using the Pseudo First Order^32^ and the Pseudo Second Order as given in Eqs. (2) and (3)^33^, respectively.

The Pseudo First Order

2 $$\text{ln }(\text{qe }-\text{ qt})=\text{ln qe} - {\text{k}}_{1}\text{t}$$

The Pseudo Second Order

3 $$\frac{{\text{t}}}{{{\text{qt}}}} = \frac{1}{{{\text{k}}_{2} {\text{q}}_{{\text{e}}}{^{2} }}} + \frac{1}{{{\text{q}}_{{\text{e}}} }}{\text{t}}$$

where q_e_ is adsorption capacity at equilibrium (mg/g), q_t_ is adsorption capacity at time t (mg/g), k_1_ and k_2_ as rate constant price/slope (per minute) and t as contact time (minutes).

### Characterization of the catalyst

Nitrogen adsorption–desorption analysis was performed on a Quantachrome Autosorb Automated Gas Sorption System, with an outgas temperature of 300 °C and a bath temperature of 77 K. Analysis of the N_2_ adsorption data was performed using Autosorb for windows 1.2 provided by Quantachrome Co. The surface area was calculated through a multipoint BET model using the P/P_o_ ranging from 0.05 to 0.2. The Pore size distribution was calculated using a BJH model; and the total pore size was calculated in terms of the maximum P/P_0_ point. The crystalline phase was investigated using Philips X’pert XRD instrument with Cu Ka radiation with a step size of 0.04° and counting time of 10 s. The data were recorded in the 2-theta range in small angle 0.1–5.0° and wide angle of 5–80°. The morphology and element of sample was monitored by the micrograph of Scanning Electron Microscopy (SEM- EDX ZEIS EVO MA 10) and coated by Pd/Au and Transmission Electron Microscopy (TEM HT7700, 120 kV). The analysis of functional group conducted at range 400–4000 cm^−1^ by Fourier transform infrared spectroscopy (FTIR Shimadzu Spectrometer 2800).

## Results and discussion

**Figure 1:**
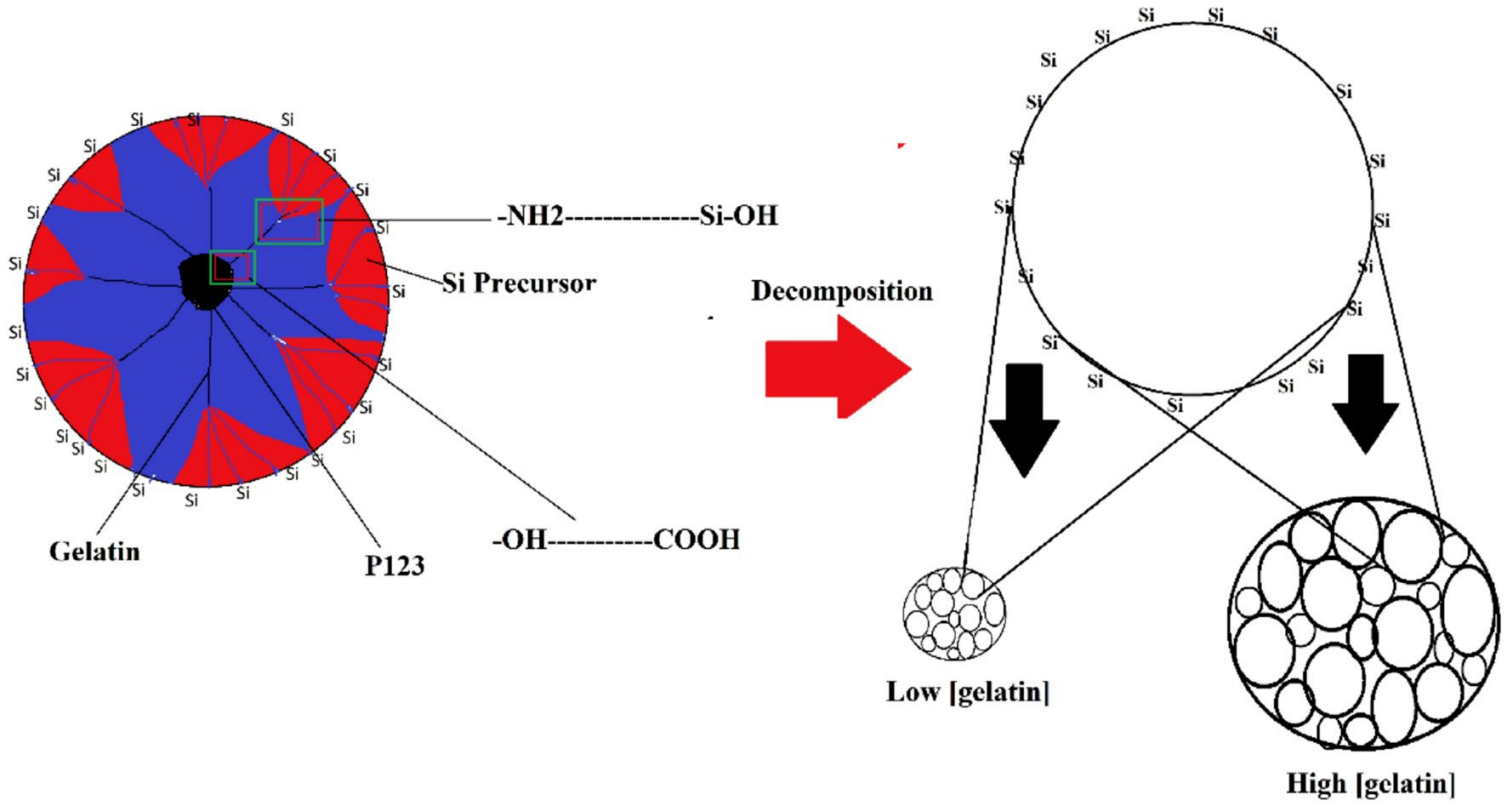
The micellar aggregation from P123-gelatin with Si precursor for NSG formation.

Mesoporous silica synthesized in the presence of gelatin showed the enlargement of mesopore diameter and particle size originated from the formation of stable micellar network between gelatin and P123 block copolymer (Fig. 1). Tetraethyl orthosilicate as silica source is spontaneously hydrolysed when added in water to form SiO_2_. However, the presence of P123 surfactant, displaced C_2_H_5_O^−^ on SiO_4_^−^ with the triblock PEO copolymer preventing the hydrolysis to SiO_2_. The presence of gelatin further stabilised the PEO structure via interaction between the –COO^−^ fragments in gelatin with –OH in PEO to form ester linkage. The –NH_2_ tail on the gelatin was protonated in acidic solution during the synthesis, consequently transformed the hybrid gelatin-P123 to anionic surfactant with positive charges on the gelatin backbone. The positive charged of gelatin was suggested to promote the elongation of the micellar structures^11,30,31,34^ Apart from that, the swelling effect of gelatin in water which enhanced at a higher gelatin concentration^35^ expanding the spacing between SiO_4_^-^ containing micelles, consequently increased the size of silica. Decomposition of gelatin and P123 molecules at high calcination temperature, produced a well-defined mesopores. The strong interaction between silicate-gelatin-P123 networks produced a stable composite, therefore calcination at high temperatures maintained the parallel pore network resulting in the pore expansion on silica^30,36^.

### SEM analysis

**Figure 2:**
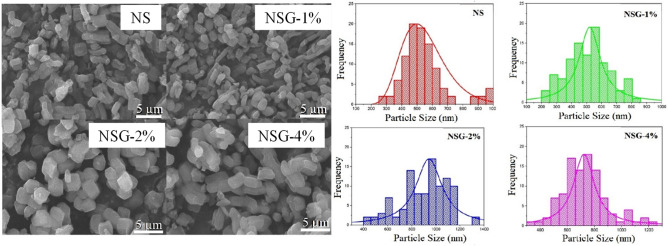
SEM analysis and the particle size distribution histogram of samples.

Figure 2 showed the SEM analysis of silica obtained using P123 (NS) and double template of P123-gelatin (NSG) at different weight ratios. In general, all silica samples showed the formation of elongated cube-like structure with the size increased at higher gelatin weight. Silica synthesized using P123 as template showed the formation of relatively small structure with the average particle size was estimated within 4–5 µm. Following the addition of 1% of gelatin during the synthesis, there is a no significant differences on the morphology, but the particle sizes were slightly increased. When the silica was synthesized using a higher concentration of gelatin at 2% and 4%, a larger elongated cube-like particles were produced with the average sizes were determined at 6–9 µm. The morphology analysis using SEM revealed that the addition of gelatin in the synthesis mixture managed to maintain the regularity of the three dimensional morphologies of the silica. However, the increase of gelatin concentration shifted the particles size distribution from 4 to 9 µm. The histogram of particle size distribution, determine based on the length of the elongated cube revealed the enlargement of particles size with increasing the amount of gelatin in the reaction mixtures.

### TEM analysis

**Figure 3:**
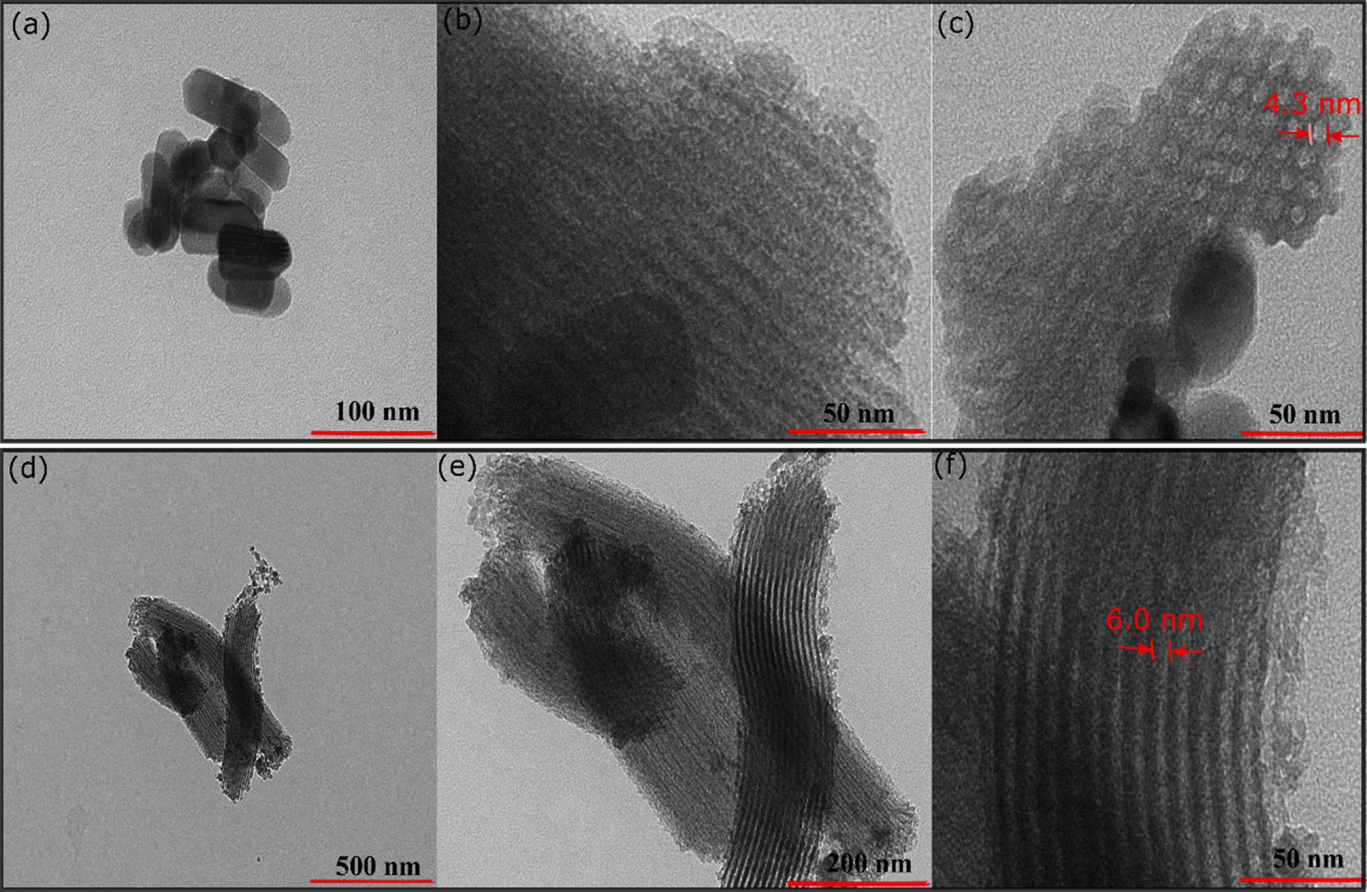
TEM analysis of NS (**a**–**c**) and NSG-1% (**d**–**f**).

TEM analysis of mesoporous silica produced using P123 and mesoporous silica from the mixture of P123 and 1% gelatin were carried out to provide high resolution morphology analysis. Figure 3a–c showed the TEM analysis of mesoporous silica produced using P123 without the presence of gelatin at different magnification. Although SEM analysis indicated the size of mesoporous silica was determined at ~ 4–5 um, the TEM analysis also revealed the formation of silica nanoparticles at ~ 100 nm size with a well-defined morphology (Fig. 3a). At much higher resolution, the presence of mesoporous channels was observed mainly on a larger silica particle (Fig. 3b). TEM analysis also revealed the average diameter of mesopores at ~ 4.3 nm (Fig. 3c). Mesoporous silica obtained using 1% gelatin and P123 mixture showed the formation of a well-defined mesoporous channel on each particle. The presence of gelatin produced mesoporous silica with larger particles size. The mesoporous channels were parallel and closely packed together forming a honeycomb structure with estimated diameter of 6.0 nm (Fig. 3d–f).

### N_2_ adsorption–desorption analysis

**Figure 4:**
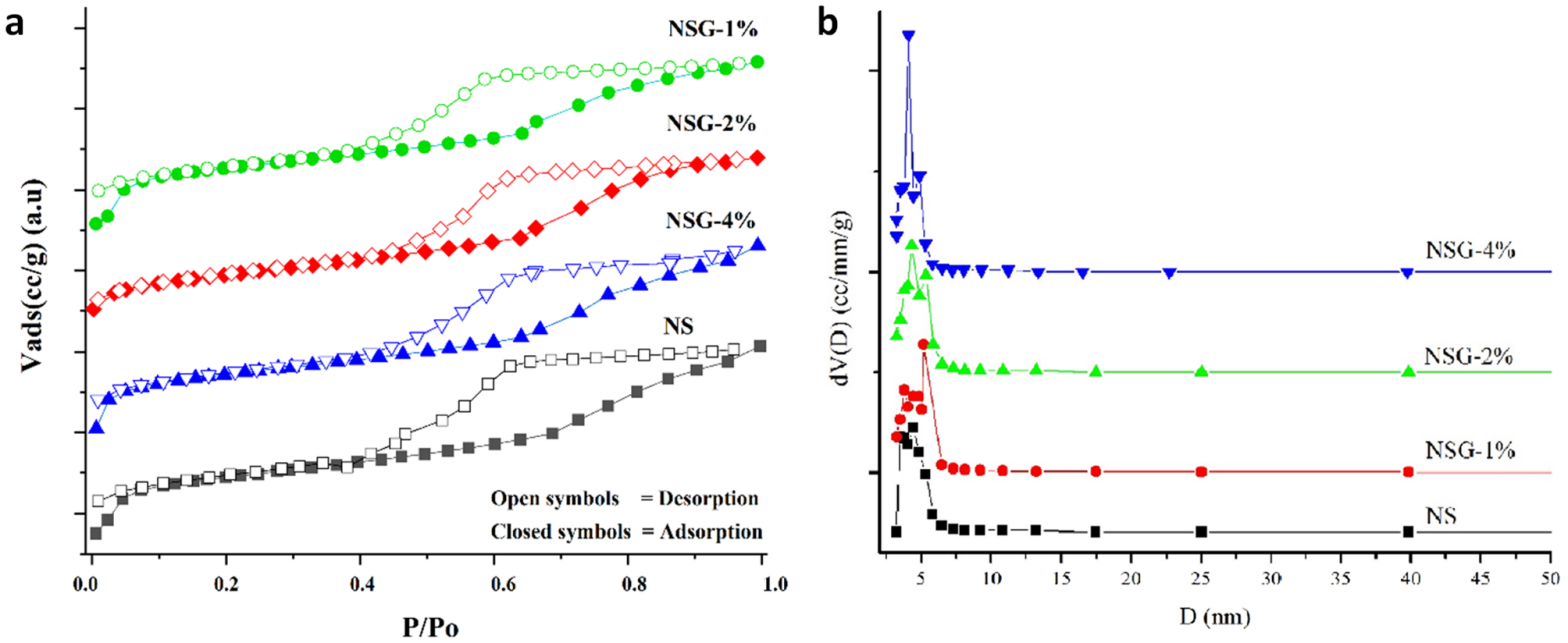
N_2_ adsorption–desorption isotherm (**a**) and Pore size distribution (**b**) of samples.

The N_2_ adsorption–desorption isotherm and the pore size distribution of mesoporous silica was depicted in Fig. 4a,b. The isotherm plot (Fig. 4a) revealed the type IV features which ascribed to the formation of mesoporous material. The presence of hysteresis loop at P/Po 0.4–0.8 indicating a low level of regularity in the structure^29,37^. The surface area of mesoporous silica produced using P123 was determined at 484.05 m^2^/g. The presence of gelatin significantly enhanced the surface area of mesoporous silica to 528.82 m^2^/g at 1% gelatin concentration. The pore volume was also improved to 0.646 cc/g compared to only 0.537 cc/g for P123 silica (Table 2). Further increased of gelatin loading to 2% and 4% slightly reduced the surface area and the pore volume, however the values were still higher than silica without gelatin. The pore size distribution of mesoporous silica obtained using P123 showed a wide pore size distribution within 2–20 nm of diameter (Fig. 4b). There is an apparent enlargement of pore diameter from 2–10 nm on to 2–14 nm with introduction of gelatin in the synthesis mixtures. Figure 4b also showed a nearly identical wide bimodal pore size distribution with two clearly peaks for all the samples. For each radius size, the increased of nitrogen uptake was observed as the concentration of gelatin increased. The observation implied the role of gelatin to increase the pore size and pore volume of mesoporous silica. It is estimated that with only 1% addition of gelatin in the synthesis mixture, contributed to the expansion of pore diameter by ~ 1–2 nm. The same phenomenon was also reported by previous researchers while using trimethylbenzene as pore expander in the synthesis of mesoporous silica nanoparticles^36^.

**Table 2:** Textural properties of samples.

Sample	S_BET_ (m^2^/g)^a^	S_micro_^b^ (m^2^/g)	V_total_ (cc/g)	V_micro_ (cc/g)^b^	V_meso_ (cc/g)^c^	D (nm)^c^
NS	484.05	75.08	0.599	0.062	0.537	6.66
NSG-1%	528.82	50.23	0.685	0.034	0.646	7.14
NSG-2%	512.21	52.38	0.676	0.038	0.638	7.14
NSG-4%	479.10	31.04	0.626	0.033	0.593	6.55

^a^S_BET_ (surface area) by BET method, measured at P/P0 < 0.3

^b^S_micro_ and V_micro_ by t-plot method.

^c^S_meso_ and D_meso_ by DFT method.

### FTIR analysis

**Figure 5:**
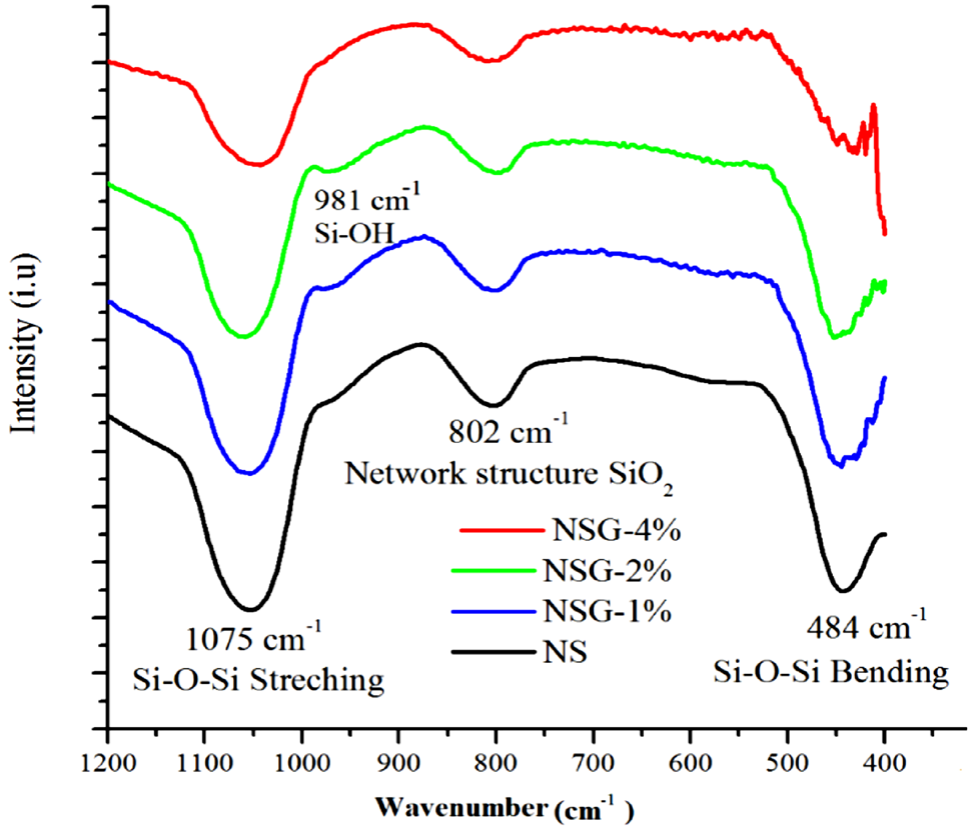
FTIR analysis of samples.

The functional group of mesoporous silica was determined using FTIR analysis shown in Fig. 5. All samples showed absorption bands at 1075 cm^−1^, 981 cm^−1^_,_ 802 cm^−1^ and 484 cm^−1^ which corresponded to Si–O–Si symmetric stretching vibration, stretching Si–OH, network SiO_2_ and Si–O–Si bending, respectively. Si–O–Si symmetric stretching vibrations at 1075 cm^−1^ showed the reduction of intensity following the use of high percentage of gelatin. The reduction of peak intensity indicated the decrease of regularity in silica crystalline framework^18,34,38^. This observation is accompanied with the reduction of network SiO_2_ band at 802 cm^−1^. Meanwhile, the Si–OH peak at 981 cm^−1^ showed the increased of intensity in silica synthesized using 1% and 2% of gelatin concentration. The emergence of Si–OH suggested the strong interaction of gelatin with Si precursors in water, creating the swelling effect which is responsible for the expansion of mesopore. TEOS is spontaneously precipitated when dispersed in water to form silica particles. The presence of gelatin allowed the formation of Si–O–Si sol stabilized by P123-gelatin hybrid. The gel is predominantly contained the Si–O–Si network^30,31,39^. Calcination at 500 °C removed the P123-gelatin templates, thus leaving the Si–O dangling bond to be compensated with trapped water from the gel swelling, generated terminal Si–OH bond. The results observed from FTIR analysis is crucial to provide information on the role of gelatin as spacer in reducing the growth of Si–O–Si bond network.

### XRD analysis

**Figure 6:**
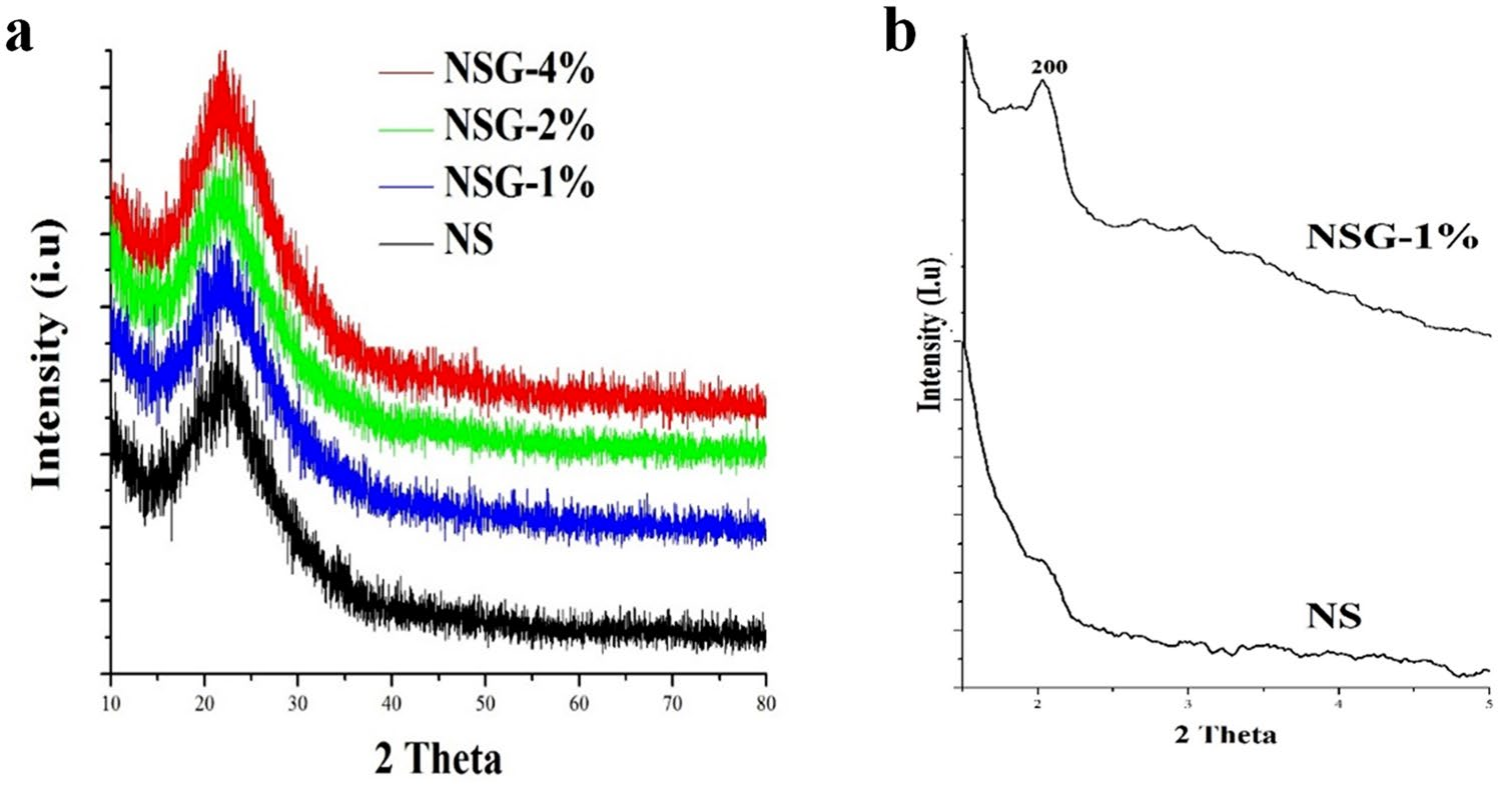
XRD analysis of mesoporous silica in wide (**a**) and low diffraction **(b**) angles.

Figure 6a showed X-ray diffractogram of silica obtained from P123 and a mixture of P123 and gelatin. All the silica samples showed similar diffractogram pattern with a broad peak centred at 2θ = 22.9° which is a typical characteristic of amorphous silica^1,37^. The intensity and the broadening of the peaks were slightly varied suggested the changes of structural properties and the crystallinity of the silica. Mesoporous silica with a lower concentration of gelatin showed a lower peak intensity as the result of smaller particle sizes. The crystalline structure of silica from P123 and a mixture of P123 and 1% gelatin were further analysed using a small angle XRD analysis in the 2-theta range of 0.5°–6° (Fig. 6b). Both silica samples showed the peak at 2.02° indexed as (200) plane, which is the characteristic of the two-dimensional hexagonal space group *p6mm*. However, the intensity of (200) plane of mesoporous silica from P123 and gelatin mixture was significantly higher than P123-derived silica. The results further confirmed the role of gelatin to increase the formation of mesopores in silica.

### Adsorption of methylene blue

**Figure 7:**
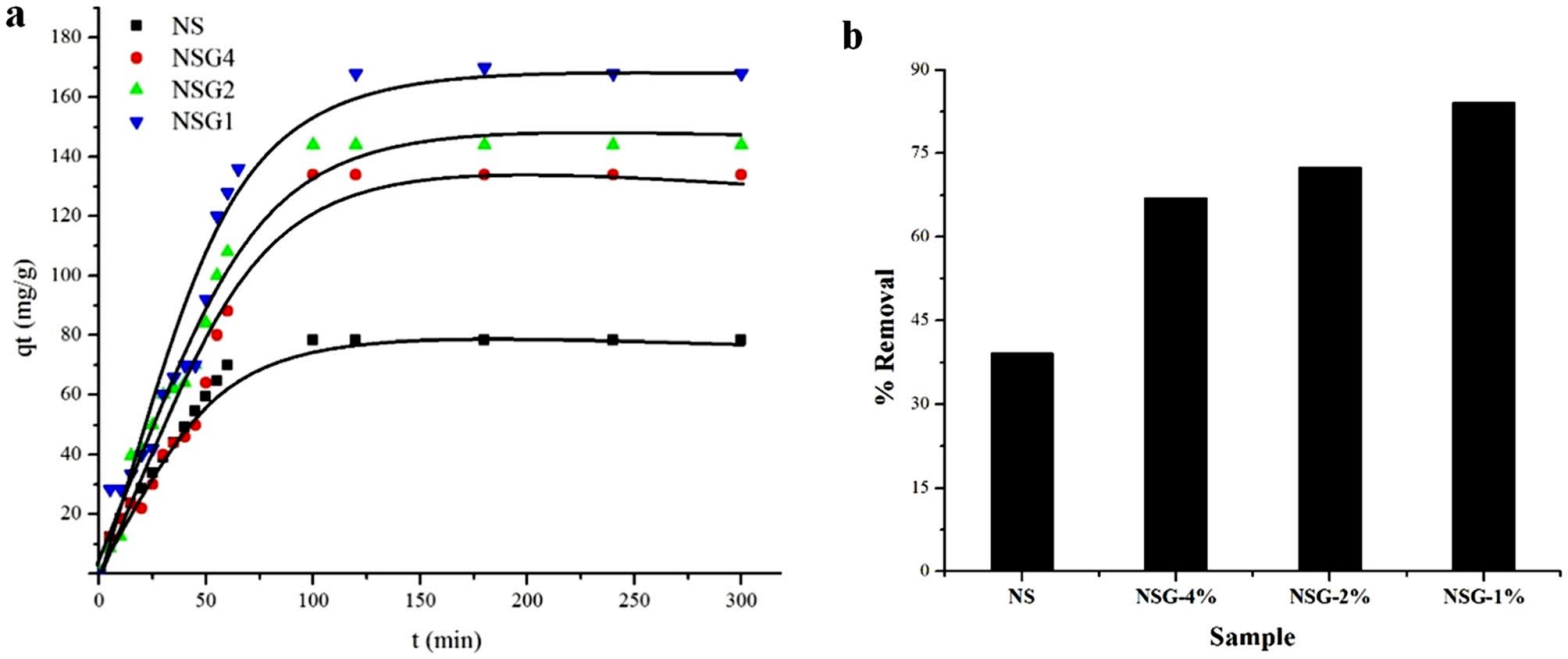
Adsorption capacity (**a**) and removal efficiency (**b**) of MB at initial concentration of 50 mg/L while using 50 mg of samples.

**Figure 8:**
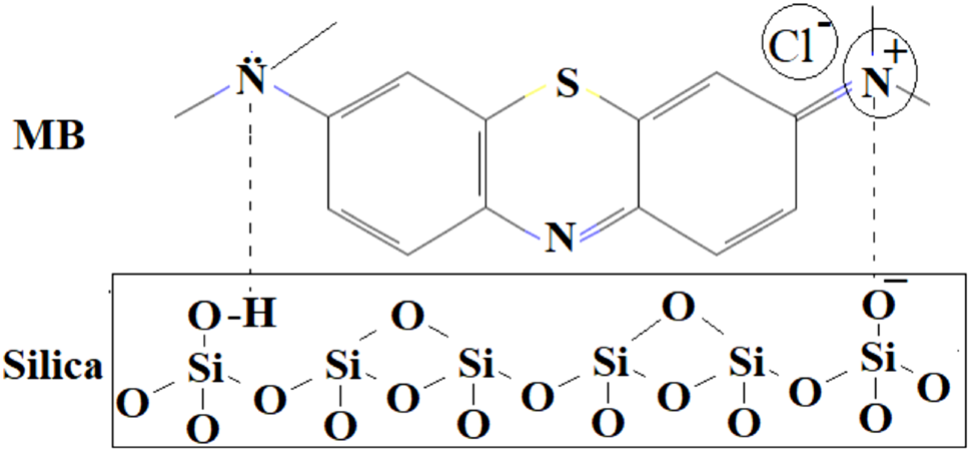
Scheme of interaction between MB and samples during adsorption.

Figure 7a showed the performance of silica as adsorbent for methylene blue removal. The adsorption results were displayed as the effect of contact time on the adsorption capacity and the removal efficiency. The adsorption capacity of the silica samples when obtained at various gelatin concentration were ranged from 130 to 170 mg/g. The adsorption plot versus contact times showed the sharp increase of MB absorption in the first 50 min before reaching equilibrium. Silica produced in the absence of gelatin only showed 78 mg/g of MB adsorption capacity, meanwhile the addition of gelatin significantly enhanced the adsorption capacity to 170 mg/g. Figure 7b showed the percentage of removal increased from 67 to 84% on the silica with increasing the gelatin concentration. The increase in the amount of methylene blue adsorption on silica produced with increasing gelatin concentration indicated that the enlargement of pore diameter is beneficial to increase MB diffusion and adsorption. Physical adsorption of MB on silica is enhanced by the high external surface area and the large mesopore diameter. Apart from that, the presence of functional group increased the adsorption of MB via chemical interaction. Silanol functional group (Si–OH) interacted with MB via the lone pair of electrons in the nitrogen atom. The interaction between methylene blue and silica NSG is described in Fig. 8. It is suggested that there are two possible interactions that occur during the adsorption of methylene blue on silica. The first possibility is the electrostatic interaction between NH_2_^+^ on methylene blue as a cationic adsorbate molecule with O^2−^ on SiO_2_ adsorbent. The second interaction occurs through hydrogen bonding between the amine groups on methylene blue with Si–OH on the silica surface. Similar mechanism on the interaction of silica and methylene blue have been proposed in several studies^10,12,40^.

**Figure 9:**
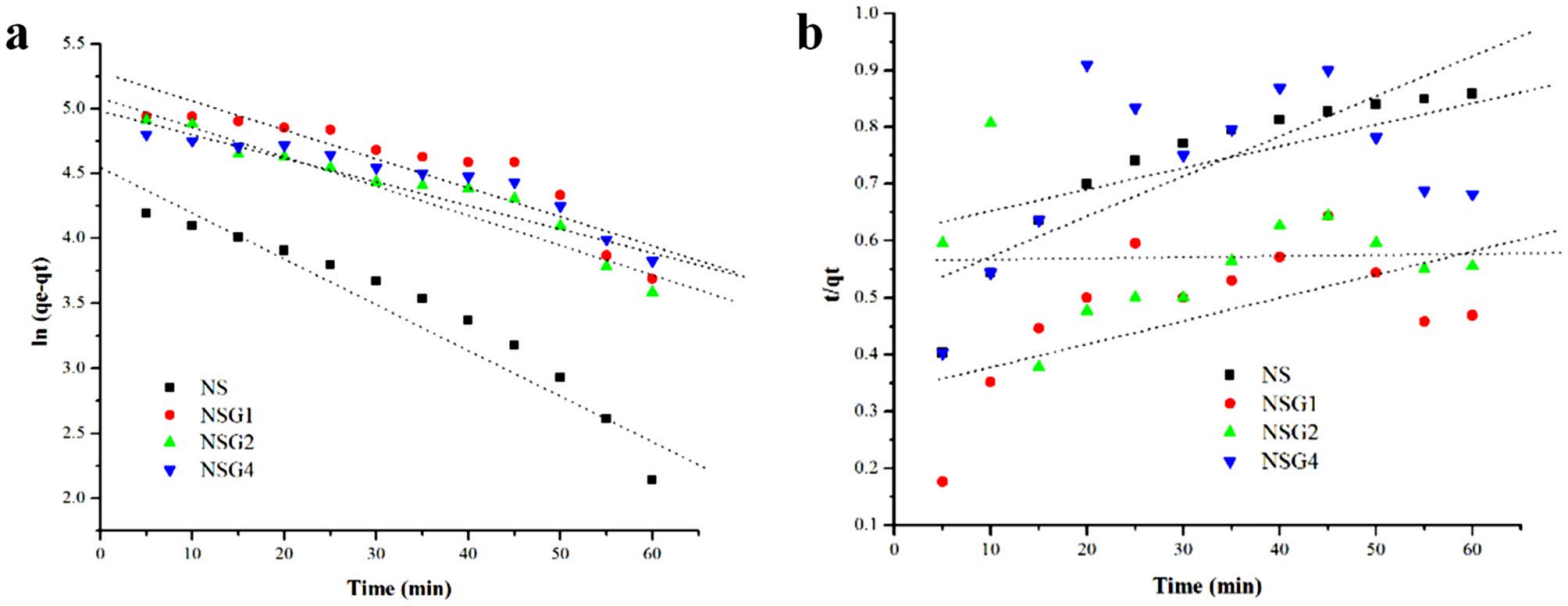
Pseudo-first (**a**) and pseudo-second-order (**b**) linear plots for the removal of MB by samples.

Based on Fig. 9a,b and Table 3, the pseudo-first-order linear plot shows a higher linearity with R^2^ values of 0.93 compared to the pseudo-second order plot. The adsorption kinetic of methylene blue on silica is in accordance with the pseudo-first-order kinetics model. The first-order pseudo adsorption kinetics means that the adsorption capacity at equilibrium is directly be the main parameter for irreversible adsorption^32^. The concentration of adsorbate is considered to have no effect on the rate of adsorption and desorption so that when the active site of the adsorbent is covered by adsorbate, the adsorption rate only depends on the difference between the adsorption capacity at equilibrium with the adsorption capacity or formulated by ${\text{q}}_{\text{e}}-{\text{q}}_{\text{t}}.$

**Table 3:** Kinetic analysis of MB adsorption using samples.

Sampel	Pseudo first order			Pseudo second order			
	R^2^	k_1_ (min^−1^)	q_cal_ (mg/g)	R^2^	k_2_ (min^−1^)	q_cal_ (mg/g)	q_e_ (mg/g)
NS	0.927	0.034	94.9	0.819	0.5023	1.99	78.36
NSG-1%	0.813	0.021	188.65	0.322	0.356	2.81	168.1
NSG-2%	0.916	0.021	159.6	0.003	0.564	1.77	144.0
NSG-4%	0.872	0.016	146.3	0.208	0.678	1.47	134.0

**Figure 10:**
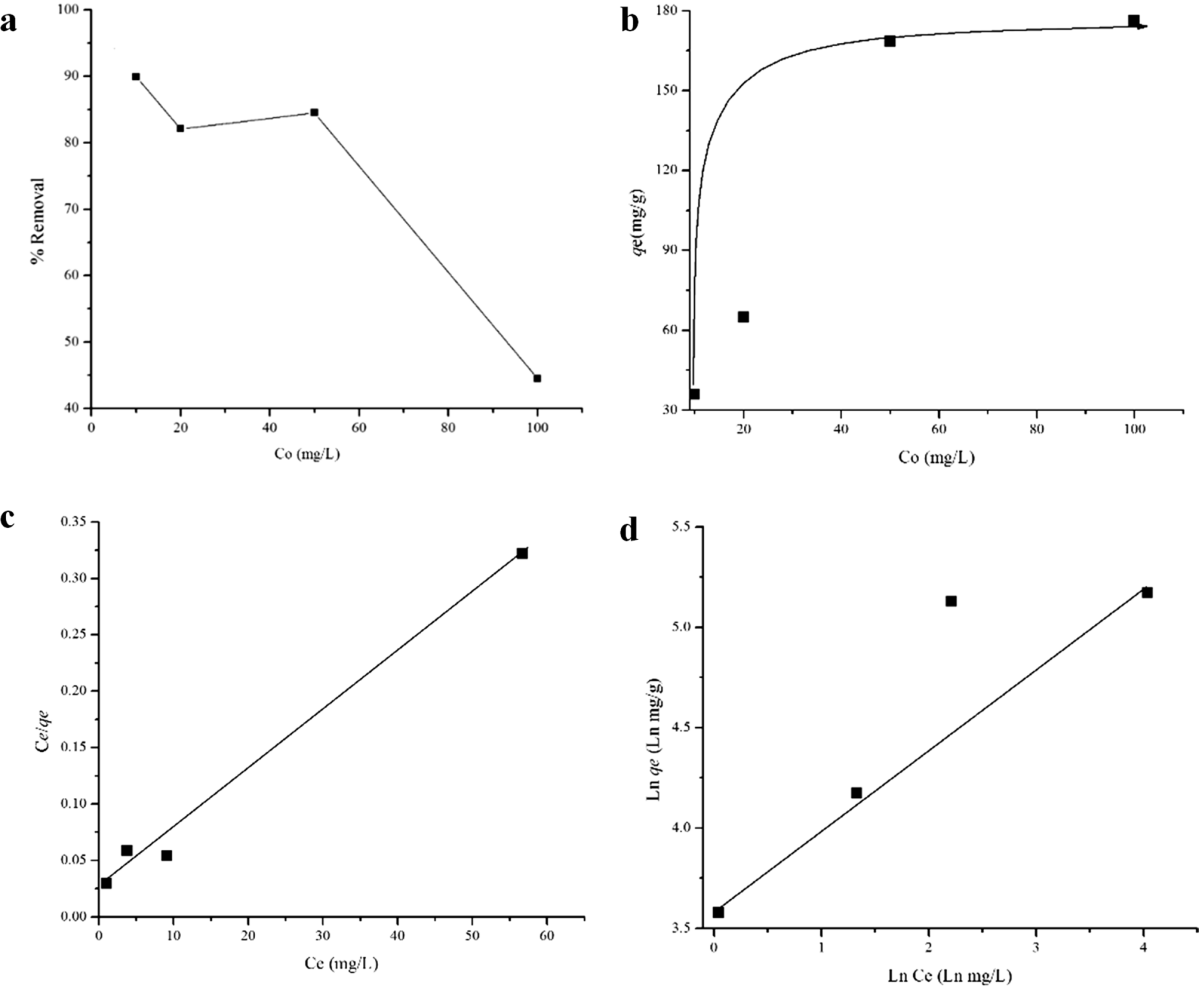
Initial concentration (C_o_) effect (**a**), %Removal (**b**), Adsorption capacity at equilibrium (q_e_) of MB fitted to isotherm of Langmuir (**c**) and Freundlich (**d**) Model.

The effect of initial methylene blue concentration on the adsorption onto 50 mg NSG is shown in Fig. 10a. The equilibrium adsorption of methylene blue (Fig. 10b) increased from 35.29 to 177.06 mg/g with the increase of MB concentrations. A high concentration of MB enhanced the diffusion and the mass transfer due to the concentration gradient that was developed between the bulk solution and the surface of adsorbent^41^. The relatively large surface area from its tubular and bimodal pore structures, as well as the presence of microporous pore walls were responsible to provide adsorption sites for a highly concentrated methylene blue solution^42^. The presence of gelatin is responsible to determine the pore characteristic and specific surface area of NSG, which in turn promotes the adsorption capacity for organic waste removal^6,43^. The equilibrium experimental adsorption data were fitted by the Langmuir and the Freundlich adsorption isotherms models to describe the mechanism of methylene blue adsorption onto mesoporous silica (Table 4). The Langmuir plot ($\frac{{C}_{e}}{{q}_{e}}$ versus ${C}_{e}$) for the adsorption data of methylene blue onto mesoporous silica fitted by a linear regression analysis (Fig. 10c). The Langmuir isotherm shows a good fit to the adsorption data with the regression coefficients (R^2^) of 0.990. The data also fitted to the Freundlich plot, with the regression coefficients (R^2^) was determined at 0.819. The values of q_max_ and k were obtained from the slope and the intercept of the linear plots. The maximum adsorption capacity (q_max_) for NSG mesoporous silica was found determined at to be 200 mg/g which may be related to the presence of more mesopores in the structure of NSG. It is known that in mesopores the dispersive forces govern the amount adsorbed due to the increased adsorption potential due to the large pore size which accessible of methylene blue molecule^12^. The results suggest that in the case of NSG mesoporous silica with its mesoporous pore wall structure^40^ the amount adsorbed is governed, at least to some extent, by the mesopores, where dispersive interactions are predominant. The Freundlich isotherm (Fig. 10d) is represented by an empirical model that describes heterogeneous adsorption and assumes that the adsorption energy decreases exponentially with surface coverage. This Freundlich afforded a lower regression coefficient than Langmuir isotherm for mesoporous silica which indicates that the Langmuir isotherm model provides the most satisfactory fit to the experimental data.

**Table 4:** Summary of experimental adsorption data fitted to the Langmuir and the Freundlich isotherm models.

Model	Formula	K	Note	R^2^
Langmuir	${q}_{e}={q}_{max} \frac{{{C}_{e}K}_{l}}{1+{{C}_{e}K}_{l}} or \frac{{C}_{e}}{{q}_{e}}=\frac{1}{{q}_{e}{K}_{l}}+ \frac{{C}_{e}}{{q}_{max}}$	0.2174 g/mg	q_max_ = 200 mg/g	0.999
Freundlich	${ln}_{{q}_{e}}=Ln Kf+ \frac{1}{n } Ln Ce$	1.312 mg/g) (g/mg )^1/n^	n = 10.4	0.819

**Figure 11:**
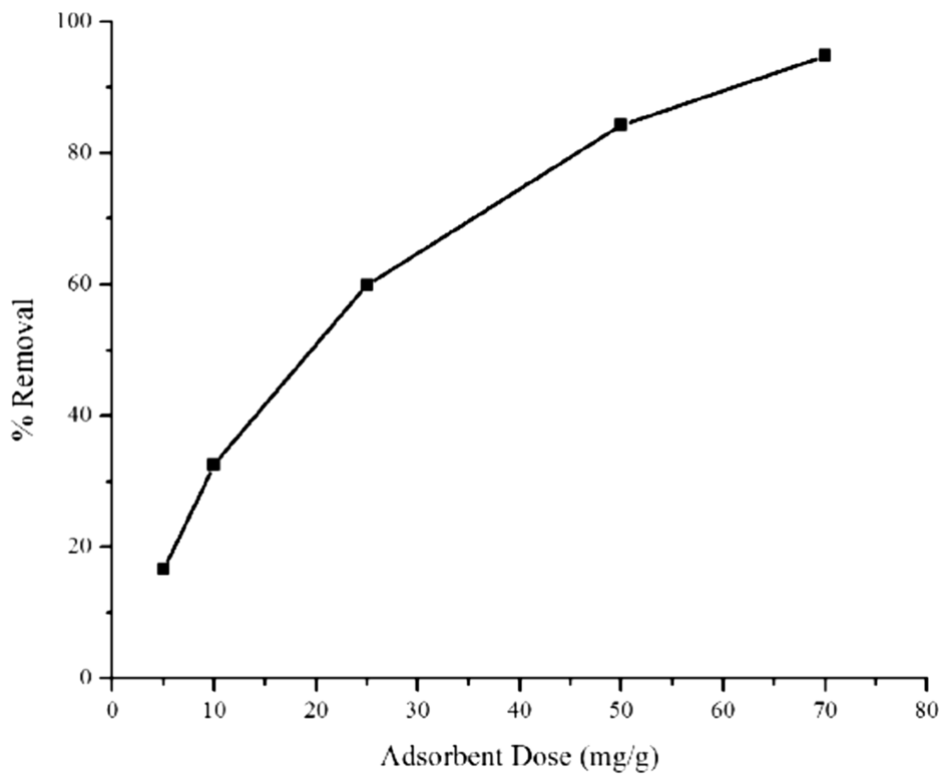
Adsorbent dose effect on %Removal of MB by NSG.

Figure 11 shows that when the amount of adsorbent was increased from 5 to 70 mg, the percentage of removal was significantly enhanced to reach 94% removal. However, Table 5 indicates that the adsorption capacity is actually reduced from 330 mg/g to only 135 mg/g when using a high amount of adsorbent. The variation of adsorption capacity at different MB concentration and adsorbent amount was associated with the efficiency of mesoporous silica in absorbing methylene blue molecules. The low initial concentration allows MB molecules to enter the pores through electrostatic and chemical interactions. The reduction of removal efficiency with increasing the initial concentration was due to the saturation of MB molecules on the pore opening that were bonded to the available hydroxyl groups. This results in the formation of multi-layer MB or pore blockage during the adsorption process^10,40^. At the beginning of adsorption until the equilibrium is reached, methylene blue will enter the pores and interact with the functional groups on the surface of silica. However, it is difficult to maintain the stability when the adsorbent amount is increased because the surface area and the pore volume were generally enhanced. Some of the pores will be occupied with MB at equilibrium, but the remaining empty sites will allow the MB molecules to desorb and diffused into the empty pores, regardless of the interaction with functional groups. This reversible adsorption process can be overcome by using the optimised adsorbent weight during adsorption. This result is in line with the previous studies which revealed that the increased weight of the adsorbent reduced the adsorption capacity, but enhanced percentage of removal especially in the high adsorbent dosage (> 20 mg)^44,45^. Although the increasing weight of adsorbent reduced the adsorption capacity, in general the efficiency reached up to 94% removal when using a high amount of silica adsorbent. The results indicate the methylene blue is efficiently removed from water in the presence of more available adsorption sites.

**Table 5:** The summary of adsorption capacity at variation of silica NSG adsorbent dosage and initial concentration of MB.

Adsorbent dose (mg)	q_max_ (mg/g)	Initial concentration (mg/L)	q_e_ (mg/g)
5	330.98	10	35.00
10	325.61	20	65.00
25	239.45	50	168.45
50	168.49	100	177.00
70	135.45	125	181.00

## Conclusion

Mesoporous silica synthesized in the presence of P123 and gelatin as green template showed the enlargement of mesopore diameter and particle size. Gelatin interacted with P123 to form extended amphoteric tail which facilitated the rearrangement of SiO_4_^-^ during crystallisation process. As the results of increased surface area and pore diameter/volume, the silica showed a higher adsorption capacity to reach 168 mg/g at the equilibrium. There is also a possibility that a higher concentration of silanol group on silica obtained using gelatin-P123 hybrid template enhanced the adsorption of methylene blue with silica via chemical interaction.

## Data Availability

The datasets used and analysed during the current study are available from the corresponding author on reasonable request.
